# Establishment and Identification of a CiPSC Lineage Reprogrammed from FSP-tdTomato Mouse Embryonic Fibroblasts (MEFs)

**DOI:** 10.1155/2018/5965727

**Published:** 2018-12-25

**Authors:** Ruiping Chen, Wenxiu Xie, Baomei Cai, Yue Qin, Chuman Wu, Wenyi Zhou, Chunhua Zhou, Shengyong Yu, Junqi Kuang, Bin Yang, Mingyi Zhao, Ping Zhu

**Affiliations:** ^1^Guangdong Cardiovascular Institute, Guangdong General Hospital, Guangdong Academy of Medical Sciences, Guangzhou, Guangdong 510080, China; ^2^School of Medicine, South China University of Technology, Guangzhou, Guangdong 510006, China; ^3^CAS Key Laboratory of Regenerative Biology, South China Institute for Stem Cell Biology and Regenerative Medicine, Guangzhou Institutes of Biomedicine and Health, Chinese Academy of Sciences, Guangzhou 510530, China; ^4^Guangdong Provincial Key Laboratory of Stem Cell and Regenerative Medicine, South China Institute for Stem Cell Biology and Regenerative Medicine, Guangzhou Institutes of Biomedicine and Health, Chinese Academy of Sciences, Guangzhou 510530, China; ^5^University of Chinese Academy of Sciences, Beijing 100049, China; ^6^Guangzhou Institute of Cardiovascular Disease, the Second Affiliated Hospital, Guangzhou Medical University, Guangzhou, Guangdong 510260, China

## Abstract

Safety issues associated with transcription factors or viruses may be avoided with the use of chemically induced pluripotent stem cells (CiPSCs), thus promoting their clinical application. Previously, we had successfully developed and standardized an induction method using small-molecule compound, with simple operation, uniform induction conditions, and clear constituents. In order to verify that the CiPSCs were indeed reprogrammed from mouse embryonic fibroblasts (MEFs), and further explore the underlying mechanisms, FSP-tdTomato mice were used to construct a fluorescent protein-tracking system of MEFs, for revealing the process of CiPSC reprogramming. CiPSCs were identified by morphological analysis, mRNA, and protein expression of pluripotency genes, as well as teratoma formation experiments. Results showed that after 40-day treatment of tdTomato-MEFs with small-molecule compounds, the cells were presented with prominent nucleoli, high core-to-cytoplasmic ratio, round shape, group and mass arrangement, and high expression of pluripotency gene. These cells could differentiate into three germ layer tissues in vivo. As indicated by the above results, tdTomato-MEFs could be reprogrammed into CiPSCs, a lineage that possesses pluripotency similar to mouse embryonic stem cells (mESCs), with the use of small-molecule compounds. The establishment of CiPSC lineage, tracked by fluorescent protein, would benefit further studies exploring its underlying mechanisms. With continuous expression of fluorescent proteins during cellular differentiation, this cell lineage could be used for tracking CiPSC transplantation and differentiation into functional cells.

## 1. Introduction

Generation of induced pluripotent stem cells was first reported by Japanese scientists Yamanaka and Takahashi for which they were awarded the Nobel Prize. In 2006, Takahashi and Yamanaka [1] first introduced four transcription factors (*Oct4*, *Sox2*, *Klf4*, *and c- Myc*) into somatic cells using viral vectors, successfully inducing the transformation of somatic cells into induced pluripotent stem cells (iPSCs). The iPSCs, obtained after reprogramming, were similar to embryonic stem cells (ESCs) with respect to morphology, gene and protein expression, epigenetic modification, cell doubling and multiplication ability, embryoid body and teratoma forming ability, differentiation ability, and many more. In the following year, human iPSCs were produced simultaneously by Takahashi et al. and Yu et al. [2, 3]. For more than 10 years, iPSCs have shown better prospects in basic research as well as novel therapies. Using different transcription factors, fibroblasts could be reprogrammed into neuronal cells [4–7], hepatocytes [8–10], cardiomyocytes [8–10], and hematopoietic cells [11], which were clinically more valuable than traditional allogeneic stem cell transplantation therapy, and global regenerative medicine research had thus entered a new era of milestones [12, 13]. However, integration of the foreign gene into the host cell genome, by means of a viral vector, could also cause safety hazards, such as overexpression of the foreign gene, thus limiting its clinical application [14].

Researchers have attempted to either replace the Yamanaka factors with small-molecule compounds or facilitate the reprogramming process. For example, vitamin C could promote somatic cell reprogramming in mice and humans [15], *RepSox* could replace *Sox2*, one of the Yamanaka factors [16] and *BMP4* could replace *KLF4* factor in the mesenchymal-epithelial transition (MET) process during reprogramming and complete the fibroblast-to-epithelial cell transformation [17]. In 2013, Hou et al. [18] replaced *Oct4* with the small-molecule compound forskolin, and for the first time, successfully reprogrammed chemically induced pluripotent stem cells (CiPSCs) from somatic cells using a small-molecule compound. Integration of the small-molecule compound into DNA could remodel the chromatin structure, thus altering gene expression, which is significantly different from what happens in traditional methods. The safety hazards and application challenges due to viruses and transcription factors could be avoided with the use of this novel method. Further studies were conducted using small-molecule compounds to directly reprogram somatic cells into neurons, neural progenitor cells [19–21], cardiomyocytes [22], and many more.

However, there are two obvious limitations of the traditional chemically induced method, including low efficiency of the reprogramming induction and the use of serum that may affect further study of the underlying mechanisms. The chemically induced reprogramming method, reported by us earlier, not only improved the induction efficiency remarkably but also did not require serum, hence confirming it as a promising method for inducing pluripotent stem cells [23].

The Cre/loxP recombination system, proposed by Sternberg and Hamilton [24], refers to the technical core of conditional gene targeting, inducible gene targeting, and spatiotemporal-specific gene targeting strategies, which are widely used in novel gene targeting [25]. Owing to its high efficiency and simplicity, Cre/loxP localization and recombination system has been effectively utilized in the deletion of specific genes, identification of gene functions, integration of foreign genes, gene capture, and chromosome engineering.

Fibroblast-specific protein 1 (*Fsp1*), also known as S100 calcium-binding protein A4 (*S100A4*), is the most specific molecular marker currently found in fibroblasts [26]. Fibroblasts may be genetically modified with the Cre/loxP system using the promoter of Fsp1 and tracked by the specificity of fluorescent protein, in order to identify the reprogram-initiating cells and track the reprogramming process. They could help in understanding the gradual loss of initial somatic cell characteristics during reprogramming and gradual acquisition of pluripotent stem cells at reprogramming endpoints, and in gaining insight into the key factors constraining the reprogramming efficiency, including the transitions of gene expression regulation and epigenetic modification involved.

In this study, we successfully established a CiPSC lineage reprogrammed from fluorescent protein-tracking FSP-tdTomato MEFs, with small-molecule compounds, ensuring the accuracy and authenticity of reprogram-initiating cells. The obtained CiPSCs were identified and analyzed in terms of morphological characteristics, self-renewal, proliferation, and differentiation potentials.

## 2. Materials and Methods

### 2.1. Mice

FSP1-Cre mice and Rosa26-tdTomato mice, pure black, were generated from C57B/6J background and were from Shanghai Institute of Materia Medica. All animal experiments were performed according to the Animal Protection Guidelines of Guangzhou Institutes of Biomedicine and Health, Guangzhou, China.

### 2.2. Cell Culture

High-glucose DMEM (Hyclone), supplemented with 10% FBS (NTC), 1× NEAA (Gibco), and 1× Gluta Max (Gibco), was used for MEF culture.

CiPSCs and mESCs were cultured in N2B27 medium containing 1× N2 (Gibco), 2× B27 (Gibco), 1× NEAA (Gibco), 1× Gluta Max (Gibco), 1× Sodium pyruvate (Gibco), 0.1 mM *β*ME (Gibco), 3 *μ*M CHIR99021 (MCE), 1 *μ*M PD0325901 (MCE), and 1000 units/ml LIF (Millipore).

The first-stage culture medium for CiPSCs (day 0–day 22) contained iCD1, 5 *μ*M BrdU (Sigma), 0.05 *μ*M AM580 (Selleck), 10 ng/ml BMP4 (RD), 5 *μ*M RepSox (ChemBest), 5 *μ*M EPZ5676 (TargetMol), 10 *μ*M Forsklin (ChemBest), 5 *μ*M SGC0946 (TargetMol), 0.5 mM VPA (Selleck), and 0.05 *μ*M DZNep (Selleck).

The second-stage culture medium for CiPSCs (day 22–day 40) contained high glucose, DMEM (Hyclone), 1× N2 (Gibco), 2× B27 (Gibco), 1× NEAA (Gibco), 1× Gluta Max (Gibco), 1× Sodium pyruvate (Gibco), 0.1 mM *β*ME (Gibco), 3 *μ*M CHIR99021 (MCE), 1 *μ*M PD0325901 (MCE), and 1000 units/ml LIF (Millipore).

### 2.3. Isolation and Culture of Embryonic Fibroblasts

After mating in the cage, the pregnant FSP1-Cre and Rosa26-tdTomato mice were sacrificed by cervical dislocation under anesthesia and the embryos were harvested at day E13.5. The head, tails, limbs, and the darker visceral parts were removed from the embryos. The residual part was washed thrice with DPBS. The tissues were minced, 0.15% trypsin added, and incubated at 37° C for 15 min for digestion. Digestion was terminated with an equal volume of culture medium containing 10% FBS. Cells were resuspended in culture medium containing 10% FBS after centrifugation and seeded into a 60 mm culture dish. The medium was refreshed every day, and passage or cryopreservation was performed when cell fusion reached 90%.

### 2.4. Cell Reprogramming

MEFs were seeded into a 12-well plate at a density of 50,000/well. After 24 h, culture medium containing 10% FBS was replaced by the first-stage CiPSC culture medium and recorded as day 0. The medium was refreshed every alternate day and replaced by second-stage CiPSC culture medium at day 22. The induction process was continued for 12–18 days.

### 2.5. Real-Time PCR

The cells were lysed with TRIzol reagent at 1 million cells/ml to extract RNA. ReverTra Ace (Toyobo) and oligo (dT) (TaKaRa) were used to produce cDNA, and Premix Ex Taq (TaKaRa) was used as a template for gene expression detection in real-time PCR. qPCR was performed with the primer sequences displayed in Table 1.

### 2.6. Immunofluorescence

Cells were cultured in a 24-well plate covered with glass slide (coated with 0.1% gelatin overnight). The cells were washed with PBS at day 3, fixed with 4% paraformaldehyde for 30 min at room temperature (about 15°C–25°C), and washed thrice with PBS. BSA (3%) and Trizon X-100 (0.2%) were mixed in 1 : 1 ratio for cell blocking and permeability. The cells were then incubated at room temperature for 1 h and washed thrice with PBS. After 2 h incubation with primary antibody, the cells were washed with PBS, incubated with the second antibody for 1 h in the dark, washed trice with PBS, incubated with DAPI diluted at 1 : 5000 for 1 min, and finally washed thrice with PBS before observing under a fluorescence microscope. The antibodies diluted with 3% BSA were anti-Oct4 (SC-5279, 1 : 200), anti-Sox2 (sc-17320, 1 : 100), anti-Nanog (BETHYL no. A300-397A, 1 : 200), and anti-SSEA1 (RD, MAB2155, 1 : 50).

### 2.7. Teratoma Detection

CiPSCs were digested with 0.05% trypsin for 3 min and counted after resuspension and centrifugation. Approximately 5 × 10^5^ cells were resuspended in 50 *μ*l Matrigel and inoculated into the subcutaneous tissue of NOD-SCID mice. After 1–2 months, the injection site was examined for tumor formation. If a tumor was formed, it was fixed with 4% paraformaldehyde after dissection and sent to the pathology laboratory of Guangzhou Institutes of Biomedicine and Health for paraffin sections and H&E staining.

### 2.8. Chimeric Mice Detection

Pregnant ICR mice at day E3.5 were subjected to cervical dislocation under anesthesia and obtained the blastocysts. The blastocysts were transferred to the micro-operation room for further use. Smooth and round CiPSCs were selected and injected into the blastocysts at a dose of 15 cells per blastocyst. After 2–3 h culture in an incubator, the blastocysts were transplanted into the uterus of female ICR mice for pseudopregnancy at day E2.5. Each mouse was transplanted with 10–15 blastocysts. The incision was closed with a suture clip and the mice were bred until pups were born.

### 2.9. Statistical Method

Statistical analyses and mapping were performed using SPSS22.0 and Prism version 6. All quantitative data are expressed as mean ± standard deviation and comparisons were made with independent *t*-test. *P* < 0.05 was considered statistically significant (^∗^*P* < 0.05; ^∗∗^*P* < 0.01; ^∗∗∗^*P* < 0.001).

## 3. Results

### 3.1. Preparation of FSP-tdTomato MEFs

**Figure 1:**
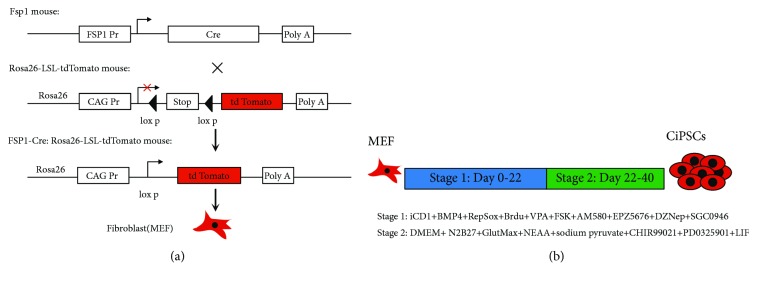
Establishment of CiPSCs derived from FSP-tdTomato MEFs. (a) Summary scheme depicting the establishment of FSP-tdTomato MEFs. FSP: fibroblast-specific protein. (b) Schematic diagram for the induction of CiPSCs from MEFs.

As shown in Figure 1(a), when the Fsp1-Cre mice were mated with Rosa26-tdTomato mice, the fibroblast FSP promoter was activated in the embryos and Cre recombinase was expressed to delete the stop sequence between the two loxP sites in the same direction, thus ensuring continuous expression of tdTomato. MEFs were labeled in this method to verify that our reprogram-initiating cells were indeed fibroblasts. Thus, tdTomato shall be continuously expressed after the cellular transition of MEFs.

**Figure 2:**
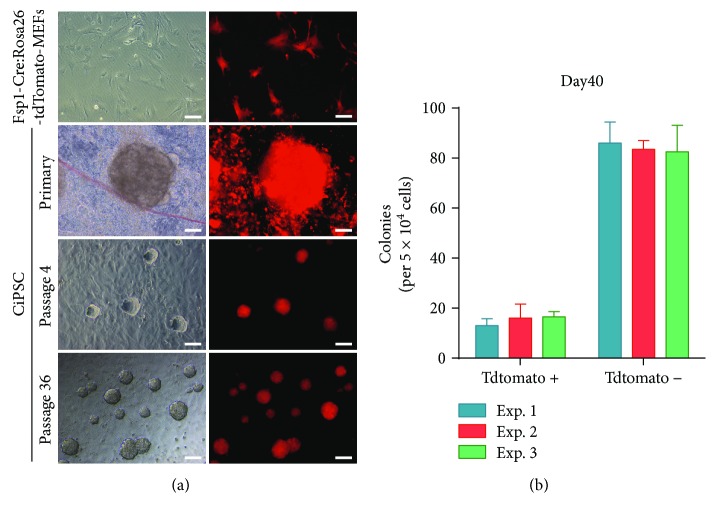
Generation of CiPSCs from FSP-tdTomato MEFs. (a) Phase and fluorescence images of FSP-tdTomato MEFs and CiPSCs. Scale bar, 100 *μ*m. (b) Quantification of CIP tdTomato + colonies generated by FSP-tdTomato MEFs. Exp: experiment. Data are mean ± SD; *n* = 2.

The MEFs isolated at E13.5 were adherent cells. Cell adherence and complete stretch were observed 2–3 h after digestion, presented as fusiform or polygonal with full cytoplasm, strong stereoscopic effect, clear nuclei, and partial expression of tdTomato red fluorescence (Figure 2(a)). On the second day of culture, numerous dead cells were found floating in the culture medium. With vigorous proliferation, numerous nascent, round, and translucent cells were observed under the microscope. Generally, cells were completely fused after 2–3 days, and passage was performed at a ratio of 1 : 2–3. MEFs at passages 3–5 that were in good condition were used for reprogramming with small-molecule compounds (Figure 1(b)).

### 3.2. Reprogramming of Fsp1-Cre:R26R^tdTomato^ MEFs into CiPSCs with Small-Molecule Compounds

Reprogramming was performed with small molecule compounds as shown in Figure 1(b). We found that tdTomato-MEFs experienced the same morphological changes as the wild-type MEFs, presenting typical clonal growth. The clones were round or elliptical with clear boundaries and good refraction. They were closely arranged, with small size, large nuclei, and the ratio of the nucleus and cytoplasm is mass (Figure 2(a)). The number of clones reached 90–108 at day 40, of which 11–20 CiPSC clones were reprogrammed from Fsp1-Cre:R26R^tdTomato^ MEFs (Figure 2(b)). The monoclonal clones of CiPSCs were selected for passage. Stable morphological characteristics, self-renewal abilities, stable proliferation, and passage were observed in the CiPSCs that had been passaged to 36^th^ generations.

### 3.3. Detection of Pluripotency Gene Expression in CiPSCs by Real-Time Quantitative PCR (RT-qPCR)

**Figure 3:**
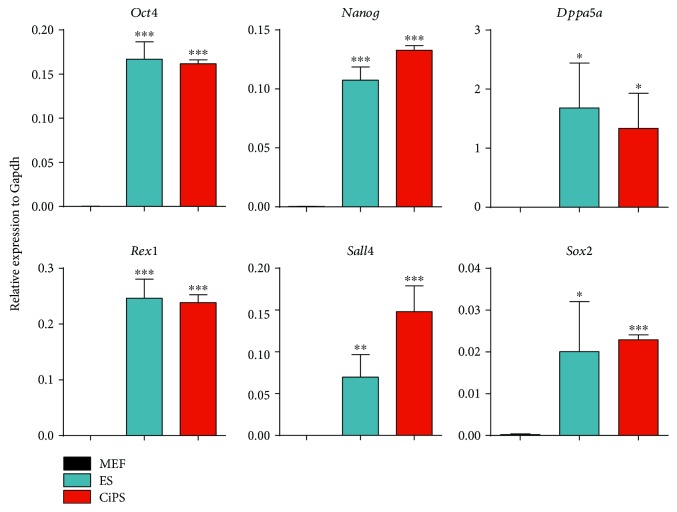
Expression of pluripotency markers in MEFs, mESCs, and three independent CiPSC lines derived from MEFs by qRT-PCR. Data are mean ± SD, *n* = 3 independent experiments. *P* < 0.05 was considered statistically significant (^∗^*P* < 0.05; ^∗∗^*P* < 0.01; ^∗∗∗^*P* < 0.001).

To understand whether pluripotency genes were activated at transcription level, Oct4, Sox2, Sall4, Nanog, Rex1, Dppa5a, and other genes were used as markers to evaluate the pluripotency of stem cells; expression of these genes was not observed in differentiated somatic cells. In this study, real-time quantitative PCR was used to analyze the expression of CiPSC-related genes. As shown in Figure 3, the pluripotency genes were hardly or rarely expressed in MEFs, while CiPSC pluripotency genes were highly expressed with mESC characteristics.

### 3.4. Immunoassay for Pluripotency Gene Expression in CiPSCs

**Figure 4:**
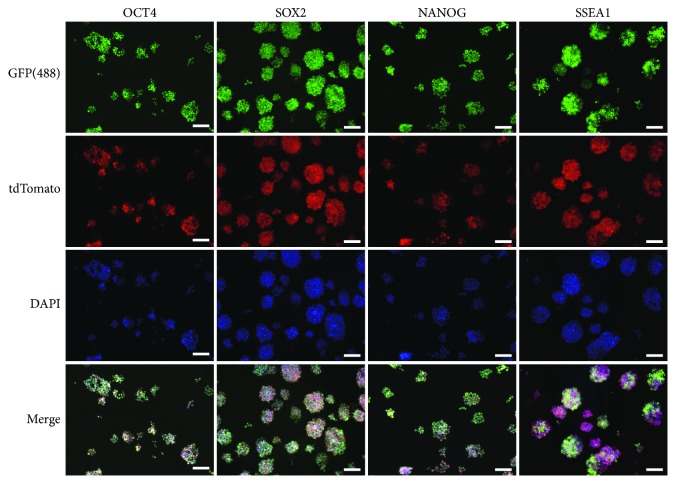
Expression of SOX2, OCT4, NANOG, and SSEA1 in MNF-derived CiPSC colonies, as detected by immunofluorescence. Scale bars, 100 *μ*m.

To further validate the expression of pluripotency genes in CiPSCs at protein level, we performed immunofluorescence assay on CiPSCs. Results showed that specific molecular markers, including ESC, SOX2, OCT4, NANOG, and SSEA1, were expressed in CiPSCs (Figure 4).

### 3.5. Teratoma Formation

**Figure 5:**
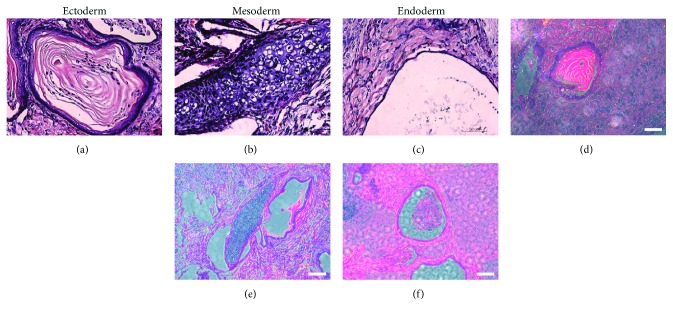
Phase (a–c) and fluorescence images (d–f) of teratoma and H&E staining of FSP-tdTomato MEF-derived CiPSCs.

To verify whether the CiPSCs were tumorigenic, they were transplanted into NOD-SCID mice. A teratoma with 2 cm diameter was formed a month after cell transplantation. H&E staining was used to further detect whether the CiPSCs had the ability to form three germ layers in vivo. The ectodermal skin tissue, mesodermal cartilage tissue, and endodermal glandular tissue were observed in the teratoma under a microscope, with most of the differentiated tissue expressed with red fluorescence (Figure 5).

### 3.6. Chimeric Mice Could Be Derived from CiPSCs

**Figure 6:**
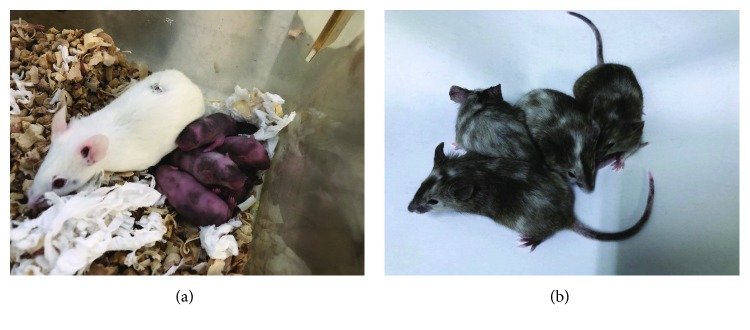
Chimeric mice generated from tdTomato-MEF-derived CiPSCs: (a) day 3 of chimeric mice and (b) 2 weeks of chimeric mice.

The cellular tissue of each organ in the chimeric mice may be derived from either the recipient blastocyst (white) or CiPSCs (black). When some cells of an organ were derived from blastocysts while others were from CiPSCs, a chimera may form. For example, if certain cells of the skin originated from blastocysts (white) while others were from CiPSCs (black), the chimeric mice would have both black and white hair. However, intersection of genome does not occur within any single cell. Chimeric mice carry cells with two different origins, each containing two different sets of genomes. However, from the perspective of hair color, the darker the hair, the greater the contribution of CiPSCs, indicating higher chimeric efficiency. Besides the skin color, we could also observe the eye color of the mice. For example, the eyes of CiPSC-derived mice should be black, while those of blastocyst-derived mice should be red. As shown in Figure 6, black eyes and hair in chimeric mice indicated chimerism from CiPSCs.

## 4. Discussion

CiPSCs were presented with two major characteristics of embryonic stem cells, namely, the self-renewal ability and pluripotency of differentiation into all types of cells. In addition, CiPSCs not only overcame the ethical issues challenged by embryonic stem cells but also could avoid the safety issues associated with viruses and transcription factors and have broad application prospects in toxicology, drug screening, disease models, and regenerative medicine [27, 28]. The earliest method for transcription-factor reprogramming was by retroviruses and lentiviral vectors, which are still widely used. Although the reprogramming efficiencies of these methods were rather high compared to that of other methods, they tend to integrate stably and randomly into chromosomes, which may increase the risk of tumorigenesis. Through biochemical tests and high-throughput screening, the effects of transcription factors could be gradually replaced by small-molecule compounds and growth factors. The main limitation that hinders the application of CiPSCs in clinical practice is efficiency and safety, and the key to solving these problems lies in the mechanistic studies underlying somatic cell reprogramming.

One remaining question is the origin of CiPSCs. It is estimated that only a small percentage of cells expressing the pluripotency genes became CiPSCs using the small-molecule compound reprogramming system. It follows that rare tissue stem/progenitor cells that coexisted in the fibroblast culture might be the reason for the increase of the CiPSCs. In fact, researchers have successfully obtained multipotent stem cells from skin [29–31]. These studies have also proved that about 0.067% of mouse skin cells are stem cells. The transformation of tissue stem cells induced by small-molecule compound may serve as a possible explanation for the low frequency of CiPSC derivation. In the current study, we constructed a fluorescent protein-tagged MEF reporter system from FSP-tdTomato mice to track the CiPSC-initiating cells and reprogrammed them into CiPSCs with a simple and efficient method using small-molecule compounds. Presented with typical mESC morphology, CiPSCs could still proliferate stably, remain undifferentiated in the 36^th^ generation, express pluripotency genes, form teratomas in vivo, and later differentiate into three germ cells. Our results indicated that the reprogram-initiating cells were accurate and authentic. The reprogrammed CiPSCs presented with the potentials of proliferation and differentiation and abilities of self-replication and renewal, which enable them to form highly differentiated functional cells with sustained fluorescent protein expression.

Furthermore, our CiPSC lineage will facilitate stem cell studies, because the bright tdtomato fluorescence would enable the visualization of the cells for long periods both *in vivo* and *ex vivo*; examples include tracing specific cell lineages derived from CiPSCs and evaluating changes of the morphology of CiPSCs. And our new CiPSC lineage may also be an ideal tool for organ transplantation research owing to improved traceability of cells and tissues [32–34]. Moreover, any tdtomato-labeled cells from CiPSCs would be traceable in the host tissue, enabling visualization of how transplanted cells/tissues behave and interact with the host.

However, the method of reprogramming with small-molecule compounds is considered to be time consuming for clinical applications. It takes about 40 days for CiPSC clone formation, whereas Yamanaka factor-mediated reprogramming takes only 7 days [35]. In a recent study, Zhao et al. [36] could reduce the reprogramming time of small-molecule compounds to 16–22 days. Theoretically, CiPSC reprogramming should be as fast as Yamanaka factor-mediated reprogramming; the discrepancy may be solved by analyzing and identifying the unique barriers during reprogramming of small-molecule compounds.

## 5. Conclusion

We successfully established a red immunofluorescence-labeled CiPSC lineage from fluorescent protein-tagged mouse fibroblasts with the Cre/loxP system. The CiPSC reprogramming method under serum-free and nonserum replacement conditions, as reported here, should be very useful for studying the underlying mechanisms of small-molecule compound-induced reprogramming and could be used as a screening platform for the barriers in the pathway. In future, we hope to establish a method of reprogramming, by small-molecule compounds, as efficient and convenient as the classical Yamanaka method. Reprogramming of human somatic cells with small-molecule compounds will be attempted in future studies, thus promoting the clinical transformation of CiPSCs.

## Figures and Tables

**Table 1:** Details of primers used for qRT-PCR analysis of selected genes used in the PCR experiments.

Gene name	Primer sequences	Annealing T (°C)	GenBank accession number
Oct4	F: 5′-CATTGAGAACCGTGTGAG-3′ R: 5′-TGAGTGATCTGCTGTAGG-3′	60°C	NM_013633.3
Sox2	F: 5′-AGGGCTGGGAGAAAGAAGAG-3′ R: 5′-CCGCGATTGTTGTGATTAGT-3′	60°C	NM_011443.4
Sall4	F: 5′-CTAAGGAGGAAGAGGAGAG-3′ R: 5′-CAAGGCTATGGTCACAAG-3′	60°C	NM_175303.4
Nanog	F: 5′-CTCAAGTCCTGAGGCTGACA-3′ R: 5′-TGAAACCTGTCCTTGAGTGC-3′	60°C	NM_028016.3
Rex1	F: 5′-CAGCCAGACCACCATCTGTC-3′ R: 5′-GTCTCCGATTTGCATATCTCCTG-3′	60°C	NM_009556.3
Dppa5a	F: 5′-CCGTGCGTGGTGGATAAG-3′ R: 5′-GCGACTGGACCTGGAATAC-3′	60°C	NM_025274.3

## Data Availability

The data used to support the findings of this study are included within the article.
